# Marginal differences in preimplantation morphokinetics between conventional IVF and ICSI in patients with preimplantation genetic testing for aneuploidy (PGT-A): A sibling oocyte study

**DOI:** 10.1371/journal.pone.0267241

**Published:** 2022-04-25

**Authors:** Neelke De Munck, Aşina Bayram, Ibrahim Elkhatib, Andrea Abdala, Ahmed El-Damen, Ana Arnanz, Laura Melado, Barbara Lawrenz, Human Mousavi Fatemi

**Affiliations:** 1 ART Fertility Clinics, Abu Dhabi, United Arab Emirates; 2 Obstetrical Department, Women´s University Hospital Tuebingen, Tuebingen, Germany; Fondazione IRCCS Ca’ Granda Ospedale Maggiore Policlinico, ITALY

## Abstract

**Objective:**

This study aimed to analyze the morphokinetic behaviour between conventional IVF and ICSI, in cycles with preimplantation genetic testing for aneuploidies (PGT-A).

**Materials:**

A randomized controlled trial (NCT03708991) was conducted in a private fertility center. Thirty couples with non-male factor infertility were recruited between November 2018 and April 2019. A total of 568 sibling cumulus oocyte complexes were randomly inseminated with conventional IVF and ICSI and cultured in an Embryoscope time-lapse system. The morphokinetic behaviour of IVF/ICSI sibling oocytes was analysed as primary endpoint. As secondary endpoints, morphokinetic parameters that predict blastocysts that will be biopsied, the day of biopsy, gender and euploid outcome was assessed.

**Results:**

When comparing IVF to ICSI, only the time to reach the 2-cell stage (t2) was significantly delayed for IVF embryos: OR: 1.282 [1.020–1.612], p = 0.033. After standardizing for tPNf (ct parameters), only Blast(tStartBlastulation-t2) remained significant: OR: 0.803 [0.648–0.994], p = 0.044. For the analysis of zygotes that will be biopsied on day 5/6 versus zygotes without biopsy, only early morphokinetic parameters were considered. All parameters were different in the multivariate model: ct2: OR: 0.840 [0.709–0.996], p = 0.045; ct6: OR: 0.943 [0.890–0.998], p = 0.043; cc2(t3-t2): OR: 1.148 [1.044–1.263], p = 0.004; cc3(t5-t3): OR: 1.177 [1.107–1.251], p<0.0001. When comparing the development between blastocysts biopsied on day 5 versus day 6, only three morphokinetic parameters were significant: cc2(t3-t2): OR: 1.394 [1.010–1.926], p = 0.044; ctBlastocyst: OR: 0.613 [0.489–0.768], p<0.0001 and ctExpandedBlastocyst: OR: 0.913 [0.868–0.960], p = 0.0004. Multivariate analysis of gender and ploidy did not reveal differences in morphokinetic behaviour.

**Conclusion:**

Minor morphokinetic differences are observed between IVF and ICSI. Early in the development, distinct cleavage patterns are observed between embryos that will be biopsied or not.

## Introduction

The use of time-lapse monitoring (TLM) in assisted reproductive technology (ART) was first reported more than half a century ago [1], though the true widespread implementation in IVF laboratories only happened a little over a decade ago, providing digital images of embryos at fixed time intervals, and allowing the assessment of embryos without physical removal from the incubator. It has contributed a great tool in assisted reproduction, as this technology was able to reveal the secret life of embryos during their *in vitro* development. It became evident that not all embryos follow the exact same pattern in their quest to develop to a blastocyst and also, there were many aspects of the development that were not yet fully understood, like reabsorption of fragments, direct cleavage and reverse cleavage. Logically, the additional information on preimplantation embryo development—compared to the static evaluations–has led to many new questions on how a specific development or which exact timing(s) can be used as viability markers to predict implantation or pregnancy, or even aneuploidy and gender.

Compared to the early days of ART in which multiple embryos were transferred, even in young patients, the current guidelines by ASRM and ESHRE highlight the importance of performing single embryo transfers. Consequently, the additional use of morphokinetic patterns were applied to select a single embryo from a cohort that has the highest implantation potential, pregnancy, and live birth rates [2, 3]. As reviewed by Cochrane in 2015 [4], there was no difference in clinical outcomes between time lapse and static evaluations. Moreover, prediction models for implantation should be developed in-house as they lose their diagnostic value if externally applied [5]. As implantation also depends on the ploidy status of the transferred embryo, the use of TLM has been investigated as a non-invasive tool to predict the euploid status of blastocysts. The conflicting outcomes were recently bundled in two reviews, indicating that there are no consistently identified morphokinetic parameters able to predict the euploidy status of embryos, results that are based on ICSI-generated blastocysts only [6, 7].

Differences in development between conventional IVF and ICSI have been explored in multiple studies [8–19]. The direct positioning of the sperm into the oocyte’s ooplasm during ICSI, results in a faster pronuclear formation and first mitotic division, however, these differences disappear around day 3 of development. Interestingly, the use of conventional IVF has recently been accepted as an alternative insemination method for couples undergoing preimplantation genetic testing for aneuploidies (PGT-A), as the whole genome amplification (WGA) protocol for trophectoderm biopsies is unable to amplify sperm DNA [19]. However, the analysis of the morphokinetic behaviour between conventional IVF and ICSI has not yet been explored in a PGT-A patient population. Furthermore, it is currently unknown if euploid/aneuploid blastocysts or blastocysts with a different gender develop differently between both insemination methods. Hence, the current prospective study scrutinised morphokinetic differences between conventional IVF and ICSI in an Arab patient population requesting PGT-A.

## Material and methods

Approval for this study was obtained from the Ethics Committee of ART Fertility Clinics, Abu Dhabi, UAE (United Arab Emirates) (Research Ethics Committee REFA024) and was registered at the ClinicalTrials.gov website (www.clinicaltrials.gov, trial number NCT03708991). A total of 42 couples signed the informed consent form and 30 of these were randomised following oocyte retrieval (OR). This was a secondary analysis of a previously published RCT analysing the differences in euploid outcomes between IVF and ICSI in patients with normozoospermia [19]. The aim of the initial study was twofold: (i) determine the embryo development and euploid rate between IVF/ICSI sibling oocytes of which the results were recently published [19] and (ii) find morphokinetic differences between IVF/ICSI sibling embryos with subgroup analysis for arrested embryos, day of blastocyst biopsy, euploid/aneuploid blastocysts and male/female blastocysts.

### Study design and study questions

**Fig 1:**
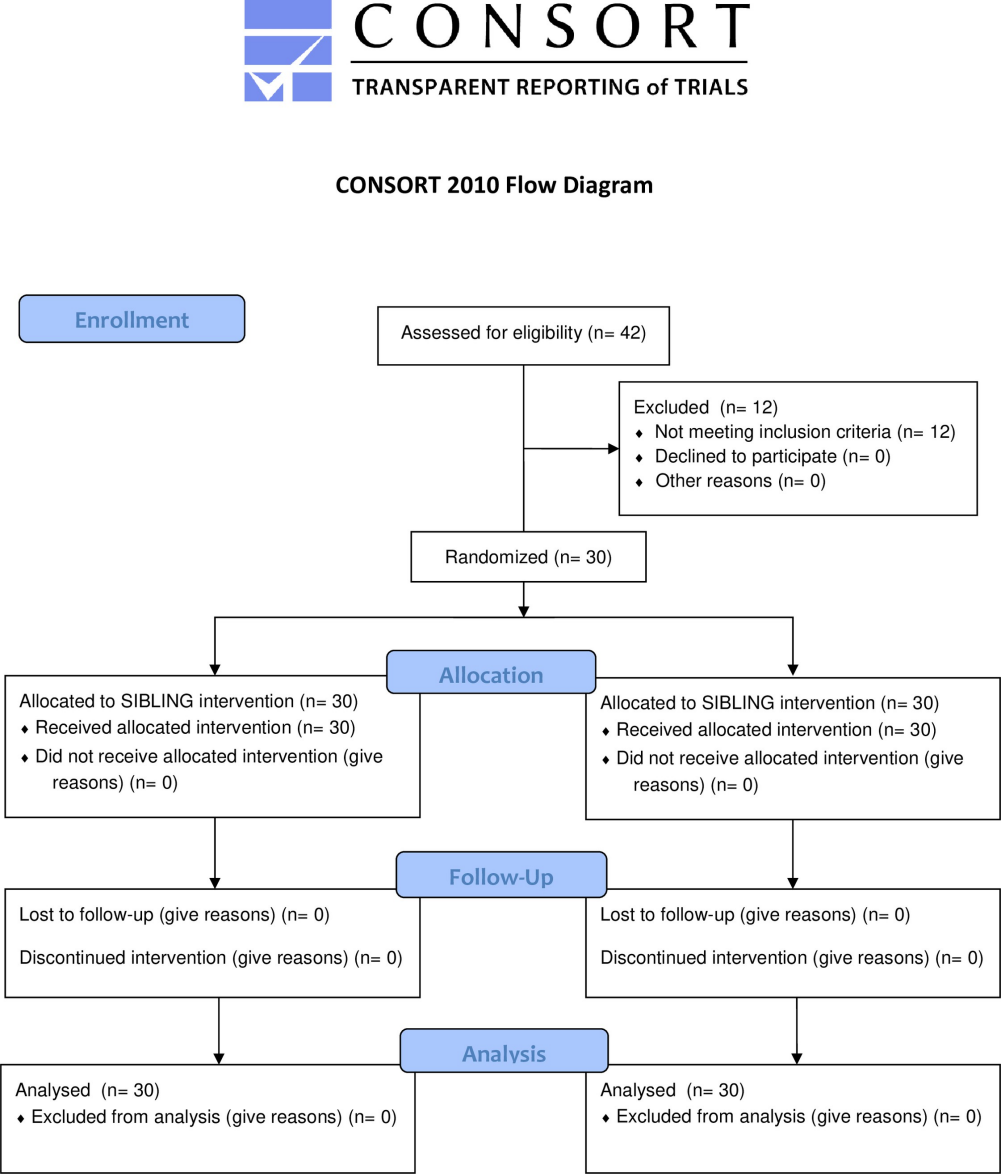
Flow chart of the enrollment and randomization of patients.

This prospective pilot study was performed at ART Fertility Clinic, Abu Dhabi, UAE, between November 2018 and April 2019. Couples had to fulfil the following inclusion criteria: female age between 18–40 years, body mass index (BMI) ≤ 30 kg/m^2^, ≥ 10 COCs after OR, antagonist protocols, Arab population, PGT-A analysis using NGS, and fresh ejaculates. Only ejaculates according to the World Health Organization [20] were eligible: < 1x10^6^/ml round cells, concentration > 15x10^6^/mL, total motility ≥ 40% and progressive motility ≥ 32%; with a progressive motility ≥ 65% after capacitation. As a preliminary semen analysis was not performed for all patients (e.g.: patients with secondary infertility), normal morphology by strict Kruger criteria was not considered. If suboptimal sperm morphology was noted on the day of OR, patients were excluded from randomisation. Every couple could only be recruited once for the study. If after the OR, at least 10 COCs were obtained, low microscope magnification was used to allocate half of these COCs to one dish (group I) and the other half of the COCs to another dish (group II). Three hours after the OR, upon denudation, an electronically generated randomisation list was opened to verify the insemination method for group I and naturally, group II received the remaining insemination method. A total of 42 couples signed the informed consent form, and 30 of these were randomised following oocyte retrieval (OR): five patients had *<*10 COCs retrieved, six patients had insufficient sperm concentration and/or motility and one patient was recruited for a different study as she experienced an IVF failure (Fig 1).

The primary objective of this secondary analysis was to detect differences in the morphokinetic behaviour of IVF/ICSI sibling oocytes. Secondary endpoints aimed to find morphokinetic parameters that predict embryo arrest (insufficient quality to biopsy), the day of blastocyst biopsy, aneuploidy and sex. In order to find embryos that will be biopsied versus embryos that will arrest, only early time-lapse parameters were considered. As arresting embryos usually fail to compact or cavitate, only parameters up until the 8-cell stage would be informative to find differences between both groups.

### Ovarian stimulation, insemination and embryo culture

The detailed protocols are described in De Munck *et al*., 2020 [19]. Briefly, standard Gonadotropin Releasing Hormone (GnRH)-antagonist-protocols were applied, using recFSH (recombinant Follicle Stimulating Hormone) or HMG (human Menopausal Gonadotropin) as stimulation medication, with a dose in accordance with ovarian reserve parameters [21]. As soon as ≥ 3 follicles ≥ 17 mm were present, oocyte maturation was triggered with 5,000–10,000 IU of hCG, 0.3 mg of GnRH agonist (Triptorelin) or dual trigger (hCG and GnRH-agonist), and OR was scheduled 36 hours later. Oocytes were collected in Quinn’s Advantage Medium with HEPES, (SAGE, Målov, Denmark) supplemented with HSA (Vitrolife, Göteborg, Sweden) (HTF-HSA), and washed in Global Total LP medium for fertilization (CooperSurgical) after which they were cultured at 37°C, 6% CO_2_ and 5% O_2_ until denudation. Insemination of both arms started 40 hours post trigger. ICSI was performed as described previously [22] and for conventional IVF, 0.3x10^6^/ml motile sperm was added to the fertilization medium and overnight incubated. After injection, oocytes were immediately cultured in Global Total medium (CooperSurgical) in the Embryoscope time-lapse incubator (Vitrolife) at 37°C, 6% CO_2_ and 5% O_2_, while IVF oocytes were inserted on day 1 after fertilization check. Embryos were cultured until blastocyst stage with medium refreshment on day 3 and trophectoderm biopsy was performed on day 5–7 of preimplantation development.

### Morphokinetic time-lapse parameters

**Fig 2:**
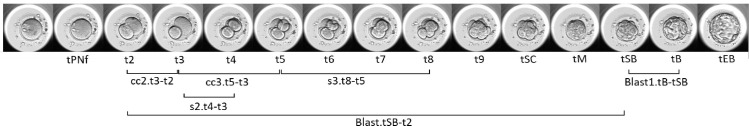
Morphokinetics timings and duration of cell cycles. Annotated time lapse parameters for all IVF and ICSI inseminated oocytes, starting from the time of pronuclear fading (tPNf), as well as variables related to the duration of specific cell cycles are depicted. t2-9: time to reach a specific cleavage stage, tSC: time of start compaction, tM: time of morula formation, tSB: time of start blastulation, tB: time to reach the full blastocyst, tEB: time to reach expanded blastocyst.

The annotation of the time-lapse parameters was performed according to the guidelines described by Ciray and colleagues [23], except for tEB (Fig 2). The morphokinetic timings for all embryos started from tPNf as, unlike the ICSI embryos who were followed from day 0, the IVF embryos were only followed from day 1 after fertilization check, a time at which both pronuclei were already visible. The following time points were carefully annotated by a single embryologist and pictures were taken every 20 minutes. tPNf: time of pronuclear (PN) fading or the first frame where both PN can no longer be visualized. t2: the time at which the first mitotic division finished and the two blastomeres are completely separated by individual cell membranes. t3-9: indicates the time to observe 3 to 9 individual blastomeres. tSC: indicates the first frame in which any sign of compaction is present. tM: marks the end of the compaction process; the morula may be fully or partially compacted. tSB: is the start of blastulation in which the cavity formation is initiated. tB: is the full blastocyst and indicates the last frame before the zona starts to thin. tEB: the fully expanded blastocyst with a thin zona. A correction was made for all TLM parameters by subtracting the time of pronuclear fading of each individual oocyte; corrected parameters are expressed as ct2, ct3 etc. Following time intervals were recorded: cc2 (t3-t2), cc3 (t5-t3), s2 (t4-t3), s3 (t8-t5), Blast (tSB-t2) and Blast 1 (tB-tSB).

### Trophectoderm biopsy and NGS analysis

Detailed protocols for TE biopsy and NGS were previously described [19]. Blastocyst biopsy was performed in 10 μl drops of HTF-HSA, the blastocyst was fixed with the holding and positioned with a clear view on the inner cell mass (ICM) at 12 o’clock, the zona pellucida was perforated by three to five laser pulses of 2.2 ms (OCTAX, Herborn, Germany). Five to ten TE cells were aspirated in the biopsy pipet followed by a mechanical “flicking” method to cut the trophectoderm cells inside the biopsy pipette, washed and placed in 0.2 ml PCR tubes containing 2.5 μL PBS and stored at -20°C until further processing.

A whole genome amplification (WGA) protocol was performed on all individual samples (PicoPlex technology by Rubicon Genomics, Inc; Ann Arbor, Michigan, USA). After WGA, library preparation consisted of the incorporation of individual barcodes for the amplified DNA of each embryo. After isothermal amplification and enrichment, sequencing was performed in a 316 or 318 chip using the Personal Genome Machine sequencing (Life-Thermofisher, USA). For sequencing analysis and data interpretation Ion Reporter software was employed. Embryos were diagnosed as euploid or aneuploid. In case of a result indicating mosaicism, the embryo was classified as “euploid” if the extent of mosaicism was below 30% and as “aneuploid” if the extent of mosaicism was above 30%. Chaotic embryos were defined as those showing a complex pattern of aneuploidies, involving more than six chromosomes. The NGS platform used herein has been validated in previous studies [24, 25] and is commercially available. Aside from the genetic outcome of the blastocyst, the sex of the embryo was also revealed.

### Statistical analysis

Continuous variables are summarized as mean and standard deviation [range]. Categorical variables are summarized as frequencies and percentages. GLIMMIX procedure was used for the univariate and multivariate analyses to consider the random effect (as one patient could have multiple embryos). Containment method was used to determine the de-nominator degrees of freedom for tests of fixed effects. The estimation technique used was Residual PL (pseudo-likelihood). With pseudo-likelihood methods, optimization begins with an initial set of pseudo-data. The response distribution chosen was Poisson and Beta with link function log and logit, respectively. The model was retained until the convergence criterion (GCONV = 1E-8) was satisfied and the estimated G matrix was positive definite. Comparisons were made using procedure PDIFF (t-test that is equivalent to the F-test) of SAS. Proc GLIMMIX was also chosen because of the capacity of handling unbalanced data. The random effect structure used for this model was Compound Symmetric (CS), also called variance components (VC). This covariance structure was chosen because the correlation does not depend on the value of lag (time distance), in the sense that the correlations between two observations are equal for all pairs of observations on the same subject. This covariance structure was chosen even though there is just one single random effect. Proc MIXED was used to analyse continuous variables. The same parameters were applied than for proc GLIMMIX. Interactions were not considered as nested factors since they were not relevant for the model. Blastocyst quality was also analysed with Proc GLIMMIX using Poisson response distribution.

P-values, Odds Ratios and Confidence Interval at 95% (OR [95%CI]) are presented in the summary tables, in association with the descriptive statistics. For the univariate analysis, a threshold of p-value <0.20 was applied to retain variables to be introduced in the multivariate model. For the multivariate analysis, a p value of 0.05 (two-sided) was considered statistically significant. To evaluate the prediction capacity of the multivariate model, a ROC curve was calculated using a logistic procedure. All analyses were performed using SAS studio (SAS® Studio). There were no missing values for any of the collected variables that were analysed.

## Results

The 568 sibling oocytes from thirty patients were randomized in this study; patients had a mean age of 30.3 ± 5.2 [22–39] years old, with a BMI of 25.1 ± 3.3 [18.8–29.9] kg/m^2^ and AMH levels of 4.2 ± 2.6 [0.85–11.68] ng/ml; further patient characteristics can be found in De Munck et al., 2020 [19]. A brief summary of the embryo development is presented in Table 1.

**Table 1:** Summary embryo development and ploidy.

	IVF		ICSI		*p value*
	n (%)	mean ± SD	n (%)	mean ± SD	
Number of COCs assigned	283	9.4 ± 4.0	285	9.5 ± 4.1	0.645
Number of mature oocytes	244 (86.2)	8.1 ± 3.7	235 (82.5)	7.8 ± 3.8	0.349
Normal fertilization	183 (64.7)	6.1 ± 3.8	190 (66.7)	6.3 ± 3.5	0.609
Blastocyst biopsy					
Day 5	80 (43.7)	2.7 ± 2.7	79 (41.6)	2.6 ± 2.1	0.941
Day 6	38 (20.8)	1.3 ± 0.8	36 (18.9)	1.2 ± 1.2	0.758
Day 7	2 (1.1)	0.07 ± 0.3	1 (0.5)	0.03 ± 0.2	NA
Total	120 (65.6)	4.0 ± 2.8	116 (61.1)	3.9 ± 2.5	0.774
Euploid blastocysts					
Day 5	43 (53.8)	1.4 ± 1.7	44 (55.7)	1.5 ± 1.4	0.923
Day 6	16 (42.1)	0.53 ± 0.7	12 (33.3)	0.4 ± 0.6	0.425
Day 7	0 (0.0)	0.0 ± 0.0	0 (0.0)	0.0 ± 0.0	NA
Total	59 (49.2)	2.0 ± 1.8	56 (48.3)	1.9 ± 1.7	0.808

Summary of embryological outcomes as presented originally in De Munck *et al*., 2020. COC: cumulus oocyte complex, IVF: in vitro fertilization, ICSI: intracytoplasmic sperm injection, NA: not applicable, n: number, %: percentage, SD: standard deviation.

### IVF versus ICSI

**Fig 3:**
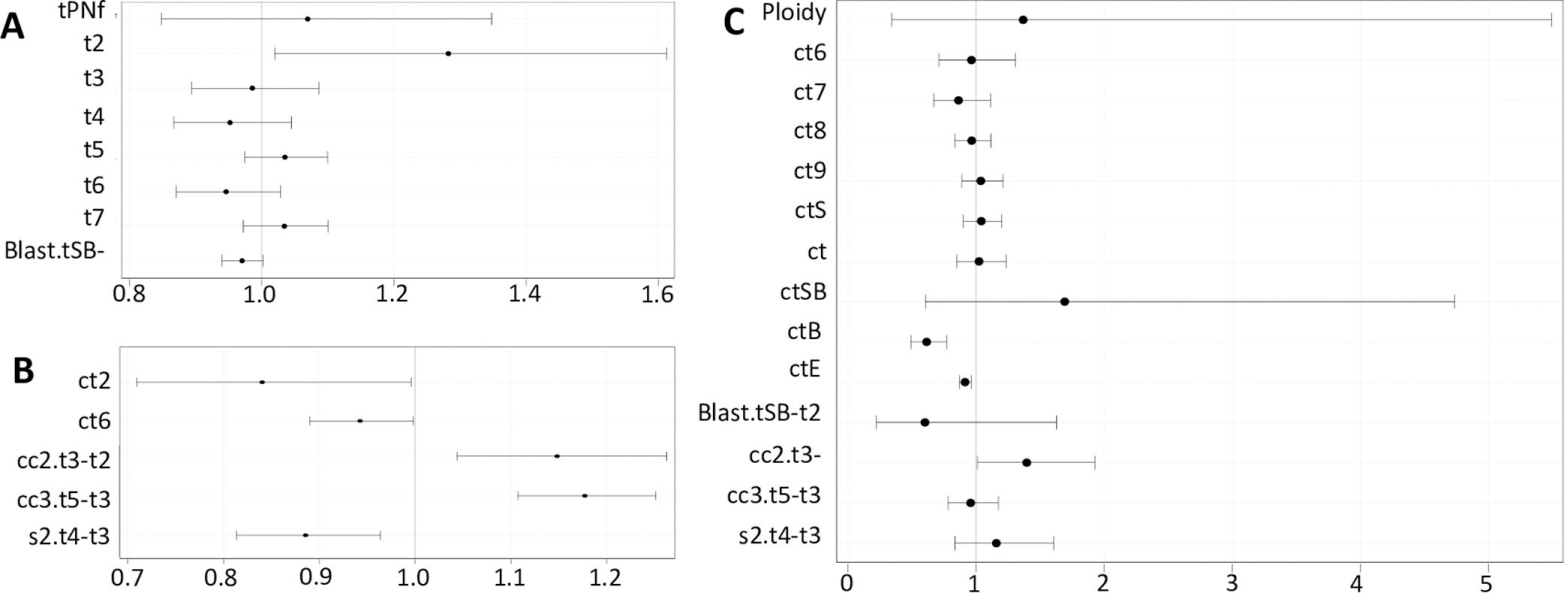
Forest plot. Odds Ratios with 95% wald confidence limits. A: IVF versus ICSI for the uncorrected values, B: blastocysts biopsied on day 5 or day 6 versus no biopsy, C: blastocysts biopsied on day 5 versus day 6.

Comparison of all TLM parameters between IVF and ICSI, for all fertilized zygotes, is presented in Table 2. Univariate analysis showed a significant delay for IVF embryos up until t7 and a shorter time between t2 and tSB (Blast). Only t2 remained significant in the multivariate model: OR 1.282 [1.020–1.612], p = 0.033 (Fig 3). After correcting for the time of pronuclear fading, only ctSB and Blast (tSB-t2) were significantly faster for IVF embryos (Table 3), though the multivariate analysis only showed a difference for Blast (tSB-t2): OR 0.803 [0.648–0.994], p = 0.004. If only biopsied blastocysts were considered, only a delayed ct2 was noted for IVF embryos; OR 1.519 [1.045–2.206], p = 0.028.

**Table 2:** IVF versus ICSI uncorrected values.

Morphokinetic parameters	IVF		ICSI		Univariate p value	Multivariate analysis	
	mean ± SD	n	mean ± SD	n		p value	OR [95%CI]
tPNf	25.9 ± 10.2	182	23.2 ± 5.9	189	**0.005**	0.569	1.069 [0.849–1.348]
t2	29.3 ± 10.4	181	25.9 ± 5.1	187	**<0.001**	**0.033**	**1.282 [1.020–1.612]**
t3	38.4 ± 10.0	180	35.4 ± 6.2	187	**0.001**	0.773	0.986 [0.894–1.086]
t4	40.2 ± 7.2	176	37.7 ± 6.7	182	**0.003**	0.302	0.952 [0.867–1.045]
t5	50.4 ± 10.7	175	46.9 ± 7.9	177	**0.001**	0.264	1.035 [0.974–1.100]
t6	53.0 ± 10.2	169	50.9 ± 8.5	174	**0.096**	0.193	0.946 [0.870–1.028]
t7	56.0 ± 9.7	163	54.1 ± 9.9	170	**0.177**	0.287	1.034 [0.972–1.100]
t8	60.8 ± 11.9	158	58.8 ± 12.5	165	0.407		
t9	70.2 ± 10.3	138	68.1 ± 11.2	149	0.437		
tSC	80.6 ± 10.9	152	77.7 ± 12.6	160	0.327		
tM	90.3 ± 10.0	147	87.4 ± 11.1	151	0.352		
tSB	98.0 ± 9.0	146	97.2 ± 10.7	149	0.496		
tB	112.3 ± 10.7	131	109.5 ± 10.8	128	0.437		
tEB	121.1 ± 10.2	99	119.4 ± 11.6	105	0.921		
Blast1.tB-tSB	15.3 ± 6.8	130	14.9 ± 6.9	126	0.969		
Blast.tSB-t2	70.8 ± 8.2	146	72.5 ± 9.7	149	**0.004**	0.064	0.970 [0.940–1.002]
cc2.t3-t2	9.1 ± 4.8	180	9.7 ± 4.2	187	0.495		
cc3.t5-t3	13.0 ± 8.8	175	12.0 ± 5.8	177	0.305		
s2.t4-t3	2.7 ± 4.9	176	2.4 ± 3.9	182	0.977		
s3.t8-t5	11.7 ± 9.9	158	11.6 ± 10.7	165	0.434		

Comparison of time lapse parameters (hours) between sibling oocytes inseminated by IVF and ICSI. SD: standard deviation, n: number, OR: odds ratio, CI: confidence interval.

**Table 3:** IVF versus ICSI standardized for tPNf.

	All fertilized zygotes					All biopsied blastocysts				
Morphokinetic parameters	IVF		ICSI		Univariate p value	IVF		ICSI		Univariate p value
	mean ± SD	n	mean ± SD	n		mean ± SD	n	mean ± SD	n	
Ploidy	NA		NA			0.50 ± 0.50	118	0.49 ± 0.50	115	0.932
Biopsy day	NA		NA			5.32 ± 0.47	118	5.31 ± 0.47	115	0.564
Sex	NA		NA			0.45 ± 0.50	108	0.52 ± 0.50	104	0.630
ct2	3.3 ± 2.5	181	3.1 ± 3.3	187	0.622	2.8 ± 1.3	118	2.5 ± 0.7	115	0.011**
ct3	12.5 ± 5.1	180	12.7 ± 5.4	187	0.915	12.9 ± 3.2	118	13.0 ± 2.5	115	0.762
ct4	15.1 ± 6.3	176	15.1 ± 5.6	182	0.798	17.8 ± 3.8	118	14.5 ± 3.0	115	0.877
ct5	25.3 ± 10.1	175	24.4 ± 7.6	177	0.369	26.5 ± 6.7	118	25.9 ± 5.2	115	0.988
ct6	28.3 ± 9.9	168	28.3 ± 7.7	174	0.737	28.9 ± 6.9	118	28.3 ± 2.8	115	0.934
ct7	31.5 ± 9.5	162	31.5 ± 8.8	170	0.623	32.1 ± 8.0	117	30.7 ± 6.9	115	0.5574
ct8	36.3 ± 11.2	158	36.3 ± 11.0	165	0.515	35.7 ± 10.5	117	34.5 ± 8.6	114	0.894
ct9	46.1 ± 9.5	137	45.9 ± 9.7	148	0.524	46.4 ± 9.1	106	45.4 ± 7.9	104	0.947
ctSC	56.2 ± 10.1	150	55.4 ± 11.9	160	0.548	55.0 ± 8.9	118	53.5 ± 9.1	115	0.721
ctM	66.2 ± 9.4	147	65.3 ± 10.5	151	0.430	64.4 ± 7.9	117	63.4 ± 8.6	114	0.816
ctSB	73.8 ± 8.3	146	75.1 ± 9.8	149	**0.009**	72.6 ± 7.3	118	72.3 ± 6.9	115	0.498
ctB	88.4 ± 10.3	131	88.0 ± 10.4	128	0.475	87.0 ± 9.3	118	86.2 ± 8.7	115	0.723
ctEB	92.5 ± 25.4	103	93.6 ± 24.7	109	0.698	92.4 ± 22.8	96	95.8 ± 15.5	101	0.226
Blast1 = tB-tSB	15.3 ± 6.8	130	14.9 ± 6.9	126	0.969	14.4 ± 5.1	118	13.9 ± 6.0	115	0.911
Blast = tSB-t2	70.8 ± 8.2	146	72.5 ± 9.7	149	0.004*	69.8 ± 7.4	118	69.8 ± 6.8	115	0.282
cc2 = t3-t2	9.1 ± 4.8	180	9.7 ± 4.2	187	0.495	10.2 ± 3.1	118	10.5 ± 2.7	115	0.258
cc3 = t5-t3	13.0 ± 8.8	175	12.0 ± 5.8	177	0.305	13.6 ± 5.9	118	13.0 ± 3.9	115	0.866
s2 = t4-t3	2.7 ± 4.9	176	2.4 ± 3.9	182	0.977	1.8 ± 3.8	118	1.5 ± 3.0	115	0.918
s3 = t8-t5	11.7 ± 9.9	158	11.6 ± 10.7	165	0.434	9.2 ± 7.8	117	8.6 ± 7.5	114	0.873

Comparison of standardized time lapse parameters (hours) between sibling oocytes inseminated by IVF and ICSI for (i) all fertilized zygotes and (ii) for all blastocysts biopsied on day 5 and day 6.

* significant difference in multivariate analysis: OR: 0.803 [0.648–0.994]; p = 0.044.

** significant difference in multivariate analysis: OR: 1.519 [1.045–2.206]; p = 0.028. Ploidy: 0 = aneuploid, 1 = euploid; biopsy day: 5 = day 5, 6 = day 6; sex: 0 = male, 1 = female. NA: Not Applicable, SD: standard deviation, n: number, OR: odds ratio.

### Blastocyst development versus embryo arrest

**Fig 4:**
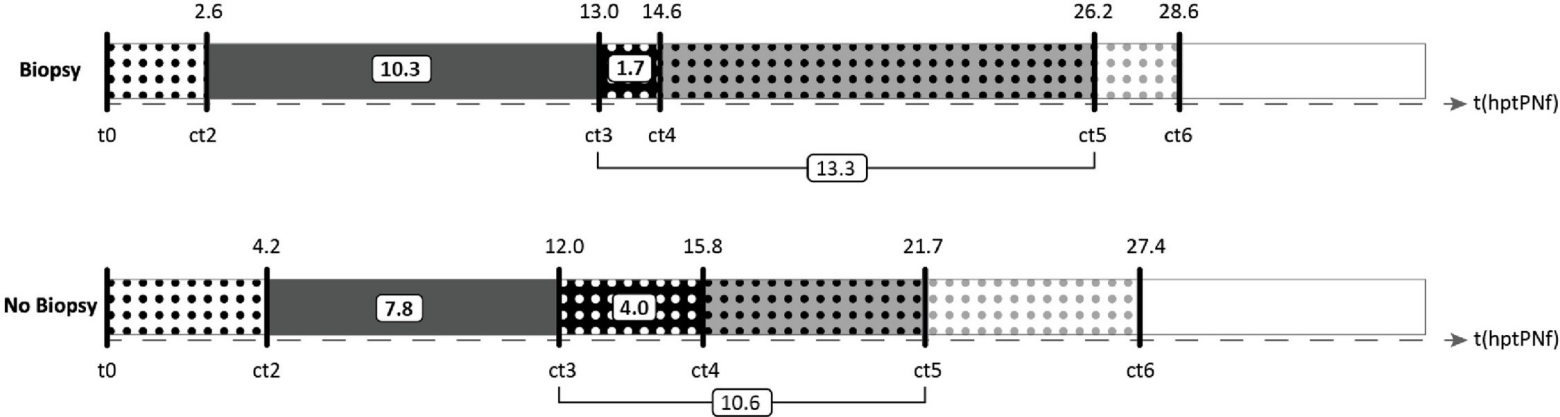
Blastocyst biopsy versus developmental arrest. Visual representation of the average TLM parameters for blastocysts with biopsy on day 5/6 versus embryos without biopsy (arrest). t(hptPNf): time hours post time of pronuclear fading. Ct2, ct3, ct4, ct5, ct6, cc2 (t3-t2), cc3 (t5-t3) and s2 (t4-t3) are displayed.

Only parameters up to ct6 were contemplated to verify very early in the development if an embryo will be biopsied on day 5/6 or if the zygote will not be biopsied (developmental arrest). Almost all analyzed parameters were significantly different between biopsied blastocysts and embryos with developmental arrest: ct2, ct3, ct5, ct6, cc2 (t3-t2), cc3 (t5-t3) and s2 (t4-t3) (Table 4). Five parameters were found to be statistically significant for embryo arrest in the multivariate model: ct2: OR 0.840 [0.709–0.996]; p = 0.045; ct6: OR 0.943 [0.890–0.998]; p = 0.043; cc2 (t3-t2): OR 1.148 [1.044–1.263]; p = 0.004; cc3 (t5-t3): OR 1.177 [1.107–1.251]; p<0.0001 and s2 (t4-t3): OR 0.886 [0.814–0.964]; p = 0.005 (Figs 3 and 4) with an AUC of 0.802. Two parameters, ct3 and ct5, were not included in the model due to collinearity with cc2 and cc3, respectively.

**Table 4:** Morphokinetic differences between embryo with and without biopsy.

Morphokinetic parameters	Biopsy Day 5 or Day 6		No biopsy		Univariate p value	Multivariate analysis	
	mean ± SD	n	mean ± SD	n		p value	OR [95%CI]
Treatment	0.51 ± 0.50	233	0.46 ± 0.50	137	0.211		
ct2	2.6 ± 1.1	233	4.2 ± 4.4	132	**0.0001**	**0.045**	**0.840 [0.709–0.996]**
ct3	13.0 ± 2.9	233	12.0 ± 7.8	131	**0.101**	ƚ	
ct4	14.6 ± 3.4	233	15.8 ± 8.6	122	0.341		
ct5	26.2 ± 6.0	233	21.7 ± 12.0	116	**<0.0001**	ƚ	
ct6	28.6 ± 6.4	233	27.4 ± 12.2	106	**0.036**	**0.043**	**0.943 [0.890–0.998]**
cc2.t3-t2	10.3 ± 2.9	233	7.8 ± 6.1	129	**<0.0001**	**0.004**	**1.148 [1.044–1.263]**
cc3.t5-t3	13.3 ± 5.0	233	10.6 ± 10.2	115	**0.0001**	**<0.0001**	**1.177 [1.107–1.251]**
s2.t4-t3	1.7 ± 3.4	233	4.0 ± 5.2	121	**<0.001**	**0.005**	**0.886 [0.814–0.964]**

Early morphokinetic parameters (hours) to detect differences between embryos with and without biopsy.

ƚ: Not Estimated due to collinearity with cc2 and cc3, respectively, SD: standard deviation, n: number, OR: odds ratio, CI: confidence interval.

### Blastocyst development on day 5 versus day 6

TLM parameters between 159 biopsied blastocysts on day 5 and 74 biopsied blastocysts on day 6 were compared. Except for ct2, ct4 and ct5, all remaining TLM parameters were significantly different between blastocysts biopsied on day 5 or day 6, as well as the ploidy status of the blastocyst (Table 5). After the multivariate analysis, only three parameters remained significantly different between day 5 and day 6 biopsied blastocysts: ctB: OR 0.613 [0.489–0.768]; p<0.0001; ctEB: OR 0.913 [0.868–0.960]; p = 0.0004 and cc2 (t3-t2): OR 1.394 [1.010–1.926]; p = 0.044 (AUC 0.978) (Fig 3).

**Table 5:** Blastocyst biopsy on day 5 versus day 6.

Morphokinetic parameters	Day 5		Day 6		Univariate p value	Multivariate analysis	
	mean ± SD	n	mean ± SD	n		p value	OR [95%CI]
Treatment	0.50 ± 0.50	159	0.51 ± 0.50	74	0.564		
Ploidy	0.55 ± 0.50	159	0.38 ± 0.49	74	**0.006**	0.660	1.366 [0.340–5.491]
Sex	0.48 ± 0.50	146	0.50 ± 0.50	66	0.761		
ct2	2.6 ± 1.1	159	2.71 ± 1.1	74	0.486		
ct3	13.1 ± 2.3	159	12.6 ± 3.8	74	**0.191**	ƚ	
ct4	14.3 ± 2.7	159	15.3 ± 4.6	74	0.391		
ct5	25.7 ± 4.5	159	27.3 ± 8.4	74	0.311		
ct6	27.5 ± 4.8	159	31.0 ± 8.3	74	**0.003**	0.810	0.963 [0.710–1.307]
ct7	29.7 ± 6.1	158	34.9 ± 8.9	74	**<0.0001**	0.254	0.862 [0.668–1.113]
ct8	32.8 ± 7.7	158	40.0 ± 11.3	73	**<0.0001**	0.630	0.965 [0.835–1.210]
ct9	44.7 ± 6.7	146	48.6 ± 11.3	64	**0.010**	0.652	1.036 [0.888–1.210]
ctSC	53.2 ± 8.0	159	56.4 ± 10.5	74	**0.028**	0.598	1.039 [0.900–1.200]
ctM	62.4 ± 7.5	157	67.1 ± 8.9	74	**0.0004**	0.820	1.022 [0.846–1.234]
ctSB	69.9 ± 5.7	159	77.9 ± 6.6	74	**<0.0001**	0.692	1.691 [0.604–4.735]
ctB	82.3 ± 5.5	159	95.7 ± 8.2	74	**<0.0001**	**<0.0001**	**0.613 [0.489–0.768]**
ctEB	88.3 ± 17.4	130	105.6 ± 18.1	67	**<0.0001**	**0.0004**	**0.913 [0.868–0.960]**
Blast1.tB-tSB	12.4 ± 3.6	159	17.9 ± 7.1	74	**<0.0001**	ƚ	
Blast.tSB-t2	67.3 ± 5.8	159	75.2 ± 6.6	74	**<0.0001**	0.315	0.600 [0.221–1.626]
cc2.t3-t2	10.5 ± 2.2	159	9.9 ± 4.1	74	**0.132**	**0.044**	**1.394 [1.010–1.926]**
cc3.t5-t3	12.6 ± 3.1	159	14.7 ± 7.5	74	**0.038**	0.672	0.957 [0.781–1.173]
s2.t4-t3	1.2 ± 2.4	159	2.7 ± 4.9	74	**0.056**	0.383	1.157 [0.833–1.607]
s3.t8-t5	7.1 ± 6.3	158	12.7 ± 8.8	73	**<0.0001**	ƚ	

Comparison of standardized time lapse parameters (hours) between blastocysts biopsied on day 5 or day 6. Treatment: ICSI = 0, IVF = 1; ploidy: 0 = aneuploid, 1 = euploid; sex: 0 = male, 1 = female.

ƚ: Not Estimated due to collinearity, SD: standard deviation, n: number, OR: odds ratio, CI: confidence interval.

### Euploid versus aneuploid blastocysts

Blastocysts biopsied on day 5 and day 6 with known ploidy outcomes were considered: 115 were euploid and 118 were aneuploid. Except for the day of biopsy, also multiple TLM parameters were different between euploid and aneuploid blastocysts, especially the ones between ct6 and ctM (Table 6). None of these parameters were significant in the multivariate model (AUC 0.639).

**Table 6:** Morphokinetics between male and female blastocysts and between euploid and aneuploid blastocysts.

Morphokinetic parameters	Ploidy					Sex				
	Euploid		Aneuploid		Univariate p value	Male		Female		Univariate p value
	mean ± SD	n	mean ± SD	n		mean ± SD	n	mean ± SD	n	
Treatment	0.51 ± 0.50	115	0.50 ± 0.50	118	0.932	0.54 ± 0.50	109	0.48 ± 0.50	103	0.630
Ploidy	NA	NA	NA	NA	NA	0.55 ± 0.50	109	0.53 ± 0.50	103	0.884
Biopsy day	5.24 ± 0.43	115	5.38 ± 0.49	118	**0.006**	5.30 ± 0.46	109	5.32 ± 0.47	103	0.761
Sex	0.48 ± 0.50	115	0.49 ± 0.50	97	0.884	NA	NA	NA	NA	NA
ct2	2.5 ± 0.7	115	2.7 ± 1.3	118	**0.071**	2.6 ± 0.9	109	2.5 ± 0.6	103	0.573
ct3	13.1 ± 2.4	115	12.8 ± 3.3	118	0.686	13.0 ± 2.7	109	12.9 ± 2.7	103	0.926
ct4	14.6 ± 3.3	115	14.7 ± 3.6	118	0.502	14.6 ± 3.9	109	14.6 ± 2.4	103	0.880
ct5	26.1 ± 6.0	115	26.3 ± 6.1	118	0.276	25.9 ± 6.0	109	26.6 ± 5.4	103	**0.155**
ct6	28.2 ± 5.9	115	29.1 ± 6.8	118	**0.049**	28.4 ± 6.3	109	28.9 ± 6.2	103	0.330
ct7	30.8 ± 7.5	114	31.9 ± 7.6	118	**0.009**	31.2 ± 7.1	109	31.7 ± 8.0	103	0.308
ct8	33.8 ± 9.5	113	36.3 ± 9.5	118	**<0.001**	34.7 ± 9.5	108	34.7 ± 9.5	102	0.541
ct9	44.9 ± 8.5	105	46.9 ± 8.5	105	**0.015**	46.0 ± 8.5	99	45.7 ± 8.5	92	0.868
ctSC	53.6 ± 9.5	115	54.9 ± 8.5	118	**0.020**	54.4 ± 8.9	109	54.0 ± 9.2	103	0.708
ctM	63.6 ± 9.0	114	64.3 ± 7.5	117	**0.039**	63.9 ± 8.9	108	63.9 ± 7.8	102	0.560
ctSB	72.1 ± 7.5	115	72.7 ± 6.7	118	0.045	72.4 ± 6.8	109	72.7 ± 7.6	103	0.964
ctB	85.5 ± 8.9	115	87. ± 9.0	118	0.006	86.4 ± 8.8	109	86.9 ± 9.3	103	0.813
ctEB	92.1 ± 18.9	98	96.2 ± 19.8	99	**0.096**	91.7 ± 23.0	93	95.6 ± 16.0	85	0.296
Blast1.tB-tSB	13.3 ± 4.9	115	15.0 ± 6.0	118	**0.056**	14.0 ± 5.0	109	14.2 ± 6.0	103	0.678
Blast.tSB-t2	69.7 ± 7.5	115	70.0 ± 6.7	118	**0.088**	69.8 ± 6.8	109	70.2 ± 7.6	103	0.978
cc2.t3-t2	10.6 ± 2.5	115	10.1 ± 3.2	118	0.321	10.4 ± 2.7	109	10.4 ± 2.9	103	0.805
cc3.t5-t3	13.0 ± 4.8	115	13.5 ± 5.2	118	**0.109**	12.9 ± 4.8	109	13.7 ± 4.5	103	**0.079**
s2.t4-t3	1.5 ± 3.3	115	1.8 ± 3.6	118	0.328	1.6 ± 3.3	109	1.7 ± 3.0	103	0.942
s3.t8-t5	7.8 ± 7.1	113	10.0 ± 8.0	118	0.002	8.9 ± 7.6	108	8.2 ± 7.6	102	0.755

Comparison of standardized time lapse parameters (hours) between (i) euploid and aneuploid blastocysts biopsied on day 5 and day 6 and (ii) male and female blastocysts. Treatment: 0 = ICSI, 1 = IVF; ploidy: 0 = aneuploid, 1 = euploid; biopsy day: 5 = day 5, 6 = day 6; sex: 0 = male, 1 = female. None of the parameters remained significant in the multivariate model. NA: Not Applicable. SD: standard deviation, n: number.

### Male versus female blastocysts

The comparison of 109 male with 103 female blastocysts, revealed a significant difference for ct5 and cc3 (t5-t3) in the univariate analysis (Table 6). However, no differences were found in the multivariate model (AUC 0.536).

## Discussion

This prospective observational study, including 568 sibling oocytes from 30 patients, explored developmental kinetics by TLM between conventional IVF and ICSI, with subgroup analysis for developmental arrested, day of blastocyst biopsy, euploid/aneuploid blastocysts and male/female blastocysts. Due to delayed pronuclear formation, IVF embryos have a delay in their first mitotic division, but progress faster to the blastocyst stage. Multiple early TLM parameters are able to predict if a blastocyst will be biopsied or not, as well as the day of biopsy (day 5 or day 6). No TLM parameter was able to predict ploidy or gender.

In couples with non-male factor infertility, it has been proven that there is no benefit of ICSI over conventional IVF [26–28]. Consequently, the comparison of embryo development between both insemination methods is not new. In cases of normozoospermia (WHO), rapid progressive morphologically normal sperm is selected during ICSI, while the zona will provide a selective barrier for abnormal sperm during IVF. This translates into equal or improved blastocyst development with conventional IVF when static evaluations are used [8, 10–12, 16–18]. The analysis of embryos at short time intervals [9] or in TLM incubators [13–15], has shown a consequent delay in pronuclear formation and first mitotic division (t2), which is in accordance with the results of the current study. The quick pronuclear formation is ascribed to the direct positioning of the sperm in the ooplasm during ICSI, leading to a faster activation of the oocyte. Strikingly, after standardizing for the time of pronuclear fading, non-concurrent results were reported. In an oocyte donation model, all differences between IVF and ICSI disappeared [14]. However, the study of Bodri and colleagues [15] showed a faster blastocyst development in IVF inseminated oocytes, which is in line with our results: the time between the first mitotic division and the time to start blastulation is significantly shorter for IVF embryos. Despite the delayed blastocyst formation after ICSI, no difference was observed in the total number of biopsied blastocysts on day 5, 6 or 7 between IVF and ICSI, highlighting the marginal time differences between both insemination methods.

The knowledge on the future development of an early cleavage embryo can guide embryologists and physicians in patient-specific treatment decisions. Most available data has focused on the prediction of top or good quality blastocysts on day 5, and multiple different absolute cleavage timings and time intervals have been linked to day 5 blastocyst formation and quality: (i) duration of first cytokinesis [29, 30], (ii) duration at 3-cell stage (s2 = t4-t3) [30], (iii) cleavage time to 7 and 8-cell stage and the relative interval from 4–8 and 5–8 cells [31], (iv) s2 (t4-t3) and cc2 (t3-t2) in combination with day 3 morphology [32], (v) cleavage synchronicity from 2 to 8 cells (CS2 = ((t3-t2)+(t5-t4)/(t8-t2)) (AUC 0.786) [33], (vi) tEB as strongest predictor (AUC 0.727) or s3 (t8-t5) as best predictor before compaction [34] and (vii) tM and s3 (t8-t5) (AUC 0.849) [35]. A combination of multiple of the abovementioned parameters, together with newly identified parameters, were also shown to be different between blastocysts biopsied on day 5 and day 6 versus arrested embryos in the current study: ct2, ct6, cc2 (t3-t2), cc3 (t5-t3) and s2 (t4-t3) (AUC 0.802), with no influence of the insemination method. It has been highlighted that parameters up until the 8-cell stage should be considered to predict blastocyst formation, as short shifts in early cleavage timings (<t5) end up with longer lags from 5 to 8 cells [33]. The current study explicitly chose parameters up until the 6-cell stage, to enable a prediction in the first two days of development, as not all embryos are capable of reaching the 8-cell stage. Conspicuously, each IVF lab can identify TLM parameters that predict the development to the blastocyst stage, but it is evident that thus far, no universally accepted algorithm is available [36].

The importance of the day of blastocyst development has extensively been studied in relation to ploidy outcomes and implantation potential. Embryos that start to blastulate on day 5 have higher euploid rates compared to embryos that start to blastulate on day 6 [37], though similar euploid rates have also been described between day 5 and day 6 blastocysts [38]. In fresh embryo transfer cycles, the stimulation induced endometrial advancement causes a superiority of day 5 blastocysts compared to day 6 in terms of pregnancy and implantation potential [39]. Though a recent meta-analysis indicated the benefit of day 5 blastocysts in both fresh and frozen embryo transfer cycles [40], the available low quality of clinical evidence still questions the superiority of day 5 blastocysts [41]. More specifically, in case of euploid frozen embryo transfer cycles, day 5 and day 6 blastocysts have shown a similar pregnancy potential [38]. Most studies that use TLM parameters to predict blastocyst development, analyze top or good quality development on day 5 [29–35], and only very limited data is available on blastocyst development between day 5 and day 6 [38]. In the latter study, only early TLM parameters (tPNf to t8) were analyzed and all of them were significantly different between day 5 and day 6 blastocysts [38]. These observations are different from the ones described in the current study in which not only early TLM parameters were evaluated, but all parameters up to tEB were considered. Additionally, none of the early parameters -as described by Kimelman and colleagues [38] were retained in the multivariate model of the current study. Interestingly, euploid status of the blastocyst was significantly different between day 5 and day 6 blastocysts in the univariate model, though this variable disappeared in the multivariate model.

Aneuploidies have been ascribed to anomalies in biological events leading to unequal chromosome distribution or incomplete DNA replication. Defective cell cycle checkpoints may be associated to shorter cycles, while activated DNA repair mechanisms may be related to prolonged cell cycles [6]. While many early morphokinetic parameters have been linked to aneuploidy, self-correction mechanisms have been described in which partial compaction and partial blastulation rescue the final embryo from aneuploid cells [42]. The association between time lapse microscopy and euploid status of cleavage stage embryos or blastocysts has considerably been explored [6, 7]. Biopsied cleavage stage embryos have shown a positive association with euploidy [43–47], in which a recurring significant TLM parameter was t5-t2>20.0 h [44], >21.5 h [46] or >21.01 h [43], and cc3 (t5-t3) >10.0 h or between 11.7–18.2 h, though all with a rather low AUC (0.63). For blastocyst biopsy, t7 and t8 have been described as early independent cleavage predictors of aneuploidy [38], while mostly blastocyst TLM parameters were significantly associated with euploid outcomes; tEB<122.2 h [48], and tSB<96.2 h and tB<122.9 h [49]. On the other hand, many other studies were unable to associate specific TLM parameters with ploidy [50–54], which is in line with the results described in the current study. Even though many significant differences were observed between euploid and aneuploid blastocysts, none of them remained significant in the multivariate model. Not unimportantly and as demonstrated previously, the insemination type (IVF or ICSI) had no effect on ploidy outcomes [19, 55, 56].

When it comes to the prediction of gender based on static parameters, many studies have been performed with non-concurring results. Mammalian male embryos have shown both faster and slower development than female embryos [57–63]. The use of TLM parameters has less substantially been used in the prediction of gender. Bronet and colleagues were able to build a hierarchical model based on s2 (t4-t3)<2 h and tM between 80.8–98.3 h, that increased the likelihood -though not significant- of selecting female embryos after cleavage stage biopsy on day 3 [64]. Another study explored TLM parameters and gender in untested blastocysts based on the gender upon live birth and concluded that female embryos are strongly associated with late expanded blastocyst TLM parameters [65]. However, as untested blastocysts were not only transferred in HRT cycles but also in natural cycles, it cannot be guaranteed that all live births were obtained from the respective transferred blastocyst or were obtained from spontaneous pregnancies. The results of the current study showed that the insemination type did not affect gender, nor was any TLM parameters able to predict gender.

Different types of TLM incubators are available on the market, each with their own specifications and limitations [6, 66, 67]. As they allow pictures to be captured on regular time intervals, these embryos are not exposed to temperature and pH perturbations as is the case with static evaluation, known to harm the embryos and their development [68]. The risks and benefits of this uninterrupted culture system have recently been summarized, warranting the need for impeccable laboratory conditions to support this type of culture system [69].

In conclusion, the results of this small prospective study showed that IVF embryos show a delay in their first mitotic division and move faster to the blastocyst stage. Early in the development and irrespective of the insemination method, a prediction can be made if an embryo will arrest or if it will be biopsied, as well as the day at which the blastocyst will be biopsied. Ploidy status and gender cannot be predicted by TLM parameters and are not affected by the insemination method. Morphokinetics do matter, however, prediction models based on individual time points are hard to standardize between different laboratories and need huge sample sizes to generate reliable results. Until today, the use of TLM will aid in reducing the time to pregnancy, by selecting the embryo/blastocyst with the highest potential, especially if a cohort of embryos is available to choose from.

## Supporting information

S1 Checklist CONSORT 2010 checklist of information to include when reporting a randomised trial*. (DOC)

S1 Protocol (DOCX)
