# Supplementary material for: Marginal differences in preimplantation morphokinetics between conventional IVF and ICSI in patients with preimplantation genetic testing for aneuploidy (PGT-A): A sibling oocyte study
Source: PLoS One. 2022 Apr 25;17(4):e0267241. doi: 10.1371/journal.pone.0267241 (PMC9037924; doi:10.1371/journal.pone.0267241)
Supplement: S1 Protocol — (DOCX) [file pone.0267241.s002.docx]

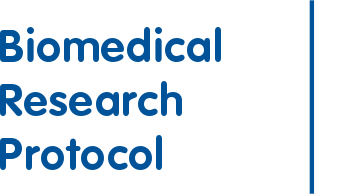


| *Effectiveness of PGT-A by using IVF versus ICSI for non-male factor infertility: a pilot study* |
| --- |
| ***1809-ABU-059-ND*** |

TEMPLATE USAGE GUIDELINES

This template can be modified according to each specific biomedical study properties.

The text is codified as:

- Fixed information goes in normal format without being colored
- Items that depend on each study and must be filled by the research team are marked as {control content field}.
- In red color are general instructions to take into consideration and help to fill this template, as a reference. They do not need to be included in the final protocol, they are simply to help the researcher complete each section. Examples of protocols already approved by an IRB can be requested from the UAGI for reference.

UAGI staff will review the protocol posting some comments, specifying in each case:

| Request | Modification | Insertion | Deletion |
| --- | --- | --- | --- |

**Warning:** The definitive text of the protocol must be in normal format and black color. This page will be deleted and it won’t be included into the final official version of the protocol.

UAGI is available to resolve any doubts and to provide support regarding the edition and development of this document.

# Project Title

Effectiveness of PGT-A by using IVF versus ICSI for non-male factor infertility: a pilot study

| Project Code: | 1809-ABU-059-ND |
| --- | --- |
|  | |
| Research Area: | Laboratory procedures and devices |
|  | |
| Date: | 12/09/2018 |
|  | |
| Keywords: | |
| IVF versus ICSI, PGT-A, Morphokinetics, Time lapse, mtDNA | |

# Sponsor information

| Name | IVI RMA Fertility, Abu Dhabi, UAE |
| --- | --- |
| Adress | Marina Village, Villa B22-23, Abu Dhabi |
| e-mail | Neelke.demunck@ivirma.com |
| Phone / Fax | +971-2-666-7049/+971-2-658-1922 |

# Principal Investigator information

| Name and surname | Neelke De Munck |
| --- | --- |
| Filiation | IVI RMA Fertility, Abu Dhabi, UAE |
| e-mail | Neelke.demunck@ivirma.com |
| Phone / Fax | +971-2-666-7049/+971-2-658-1922 |

# Responsible for the administrative management of the study

| Name and surname | Neelke De Munck |
| --- | --- |
| Filiation | IVI RMA Fertility, Abu Dhabi, UAE |
| e-mail | Neelke.demunck@ivirma.com |
| Phone | +971-2-666-7049 |

# Research team members

| **Human Mousavi Fatemi**  IVI RMA Fertility, Abu Dhabi, UAE  Human.fatemi@ivirma.com | **Ibrahim Elkhatib**  IVI RMA Fertility, Abu Dhabi, UAE  Ibrahim.elkhatib@ivirma.com |
| --- | --- |
| **Asina Bayrem**  IVI RMA Fertility, Abu Dhabi, UAE  Asina.bayrem@ivirma.com | **Ana Arnanz**  IVI RMA Fertility, Abu Dhabi, UAE  Ana.arnanz@ivirma.com |
| **Barbara Lawrenz**  IVI RMA Fertility, Abu Dhabi, UAE  Barbara.lawrenz@ivirma.com | **Neelke De Munck**  IVI RMA Fertility, Abu Dhabi, UAE  Neelke.demunck@ivirma.com |

# Responsibilities and Signatures

By signing this protocol from the project entitled: *Effectiveness of PGT-A by using IVF versus ICSI for non-male factor infertility: a pilot study*

Those undersigning state that:

- This study respects the ethical and legal rules and follows good clinical practice in its implementation
- It has the material and human resources needed to carry out the study, without interfering in other studies or clinical tasks usually entrusted to them
- They are committed that each subject is treated and controlled according the approval granted by the Ethics Committee for Clinical Research, Institutional Review Board, remaining committees and the involved authorities
- Collaborators included in this study are adequately trained for its implementation, they will have an active participation, and they consent thereto.

**Center and/or Laboratory Director**

| Dr. **Human Mousavi Fatemi**  IVI RMA Abu Dhabi Director |  | Date |
| --- | --- | --- |

**Principal Investigator**

| Neelke De Munck  IVI RMA Abu Dhabi |  | Date |
| --- | --- | --- |

# Table of contents

[Project Title 2](#_Toc525977888)

[Sponsor information 2](#_Toc525977889)

[Principal Investigator information 2](#_Toc525977890)

[Responsible for the administrative management of the study 2](#_Toc525977891)

[Research team members 3](#_Toc525977892)

[Responsibilities and Signatures 4](#_Toc525977893)

[Table of contents 5](#_Toc525977894)

[Abstract 6](#_Toc525977895)

[List of acronyms 6](#_Toc525977896)

[AIM: main objective/research question 8](#_Toc525977897)

[Secondary Objectives/research questions 8](#_Toc525977898)

[Introduction 9](#_Toc525977899)

[A. Background: 9](#_Toc525977900)

[B. Justification: 9](#_Toc525977901)

[Methodology 10](#_Toc525977902)

[A. Study Design 10](#_Toc525977903)

[B. Study period and context 10](#_Toc525977904)

[C. Reference Population 10](#_Toc525977905)

[D. Subject Inclusion/exclusion criteria 10](#_Toc525977906)

[E. Intervention and Follow-up 11](#_Toc525977907)

[Statistical methodology 11](#_Toc525977908)

[A. Data base 11](#_Toc525977909)

[B. Study Variables 12](#_Toc525977910)

[C. Sample Size 14](#_Toc525977911)

[D. Statistical data analysis 14](#_Toc525977912)

[Work plan 16](#_Toc525977913)

[Ethical issues 17](#_Toc525977914)

[Funding 18](#_Toc525977915)

[ANNEX 1. Estimation of the research Project marginal costs 18](#_Toc525977916)

[Insurance 18](#_Toc525977917)

[Publication and diffusion 18](#_Toc525977918)

[References 19](#_Toc525977919)

# Abstract

ICSI is commonly used in PGT cases to eliminate any risk of sperm DNA contamination. With the switch from cleavage stage biopsy to biopsy of the full (hatching) blastocyst, this potential risk of contamination can be neglected and could allow the use of conventional IVF in PGT cases. Especially in cases of non-male factor infertility, the use of conventional IVF can be applied as a more ‘natural’ insemination method.

A pilot study will be performed on sibling oocytes including patients with non-male factor infertility and requesting PGT-A (NGS platform). If at least 10 cumulus oocyte complexes (COCs) are obtained after oocyte retrieval, half of the oocytes will be subjected to ICSI and the other half to conventional IVF. Preimplantation development is followed by time lapse imaging and blastocysts (day 5-7) fulfilling the biopsy criteria will undergo trophectoderm biopsy to detect the ploidy state and the mtDNA copy number.

The main objective is to analyse if the use a more physiological insemination method (IVF) has a beneficial impact on the ploidy state of the blastocyst as compared to ICSI within the same patient. As the embryos will be cultured in a time lapse imaging system, annotations can be made and the differences in maturation rate, fertilization rate and embryo development between both insemination methods can be analysed as secondary outcome parameter. On top of this, the PGT-A outcome will also display the mtDNA copy number which can be compared between IVF and ICSI biopsied sibling blastocyst. Euploid blastocysts will be transferred in subsequent FET cycles and give an indication on the clinical outcome between IVF and ICSI.

Would this study enable us to undermine the dogma to perform ICSI for all PGT cycles?

# List of acronyms

| ICSI | Intracytoplasmic sperm injection |
| --- | --- |
| AMH | Anti-Müllarian Hormone |
| ASRM | American Society for Reproductive Medicine |
| BL | Blastocyst |
| BMI | Body Mass Index |
| aCGH | Array Comparative Genomic Hybridization |
| CLBR | Clinical Live Birth Rate |
| COC | Cumulus Oocyte Complex |
| DNA | Deoxyribonucleic acid |
| mtDNA | Mitochondrial Deoxyribonucleic acid |
| E_2_ | Estrogen |
| FET | Frozen Embryo Transfer |
| β-hCG | β chain of the human chorionic gonadotropin |
| hCG | Human chorionic gonadotropin |
| ESHRE | European Society of Human Reproduction and Embryology |
| IC | Informed Consent |
| ICM | Inner Cell Mass |
| IF | Impact Factor |
| IVF | In vitro fertilization |
| IU | Internal Units |
| kg/m^2^ | Kilogram per length^2^ |
| ml | milliliter |
| ng/ml | Nanogram per milliliter |
| NA | Not Applicable |
| NGS | Next Generation Sequencing |
| NP | Non-progressive motile sperm |
| OPU | Oocyte Pick-Up |
| P | Progesterone |
| pg/ml | Pictogram per milliliter |
| PGT | Preimplantation Genetic Testing |
| PGT-A | Preimplantation Genetic Testing for Aneuploidies |
| PGT-M | Preimplantation Genetic Testing for Monogenic Disorders |
| PN | Pronuclei |
| PR | Progressive Motile Sperm |
| SOP | Standard Operating Procedure |
| SIVIS | Software for medical record management |
| TE | Trophectoderm |
| UAE | United Arab Emirates |
| USA | United States of America |
| WHO | World Health Organization |
|  |  |

# AIM: main objective/research question

The primary aim is to verify if the insemination method (IVF or ICSI) on sibling oocytes influences the ploidy state of the blastocyst, as tested by PGT-A.

# Secondary Objectives/research questions

1. Pre-implantation development parameters between both insemination methods (IVF or ICSI) defined by:

- Maturation rate: number of mature oocytes obtained in each arm
  - ICSI: defined on day 0 as the number of injected oocytes/number of COCs assigned to the ICSI group
  - IVF: defined on day 1 as the number of oocytes with an extruded PB/number of inseminated oocytes
- Fertilization rate: number of zygotes with 2PN/number of oocytes assigned to the group
- Embryo quality on day 3 defined by the Istanbul consensus (Alpha Scientists, 2011)
  - The number of blastomeres and their division pattern, fragmentation, presence of compaction, vacuoles, granulation and nuclei will divide the embryos into 3 categories: good, fair or poor
  - Cell number on day 3 for all fertilized oocytes
- Embryo quality on day 5 (Gardner and Schoolcraft,1999) defined by the Istanbul consensus (Alpha Scientists, 2011)
  - The blastulation rate: number of embryos reaching at least the BL1 stage/ number of oocytes assigned to that group: defined by a 1 (yes, the embryo is blastulating) or 0 (no, the embryo is not blastulating)
  - The expansion stage of the blastocyst and the quality of the ICM and TE will divide the embryos into 3 categories
- Utilization rate: number of embryos that can be used for the patient (=number of embryos that can be biopsied)/number of oocytes assigned to the group
- Morphokinetic parameters defined by time lapse imaging starting from the time of syngamy (Basile et al., 2014)
- Morphokinetc parameters and their effect on ploidy, day of biopsy, biopsy or not and gender

1. mtDNA (de los Santos et al., 2018) per cycle and between IVF or ICSI generated embryos:

- Mitoscore value: represents the normalized mtDNA content in (an)euploid embryos and indicates the total mtDNA content in the sample

1. Cycle outcome defined by (Zegers-Hochschild et al., 2017) per cycle and also per insemination method (IVF or ICSI):

- Pregnancy (yes or no) defined by a βhCG test of > 15IU
- Biochemical pregnancy (yes or no) defined as a pregnancy in which the hCG levels start do decrease after 1 week
- Implantation rate (%) defined by the number of gestational sacs/number of embryos transferred
- Clinical pregnancy (yes or no) defined by the ultrasonographic visualization of one or more gestational sacs, including ectopic pregnancies.

# Introduction

## Background:

In case of non-male factor infertility, IVF should be chosen as insemination method. However, in Europe and the USA, 65-75% of all IVF cycles use ICSI as insemination method (Dyer et al., 2016; Boulet et al., 2015) even though there is no clear evidence why a couple would benefit from ICSI over conventional IVF as the CLBR is not different between IVF and ICSI cycles (Li et al., 2018). As many fertility clinics prefer the use of ICSI to maximize the fertilization rate, the cleavage and blastocyst formation rate is comparable when sibling oocytes are inseminated with conventional IVF or ICSI (Van Landuyt et al., 2005). Besides these static parameters, the dynamic morphokinetic parameters between such siblings have not yet been described.

If PGT is to be applied, ICSI has always been the method of choice, especially for day 3 cleavage embryos. The choice of ICSI over IVF comes from the idea that contamination of residual sperm DNA has to be prevented. With the switch of embryo biopsy from day 3 to the full (hatching) blastocyst on day 5/6, this contamination can be completely eliminated. From this viewpoint it would be interesting to analyse the ploidy outcome (PGT-A) in IVF versus ICSI cycles after trophectoderm biopsy. A similar study has been performed with the use of PGT-M on day 3 embryos (Feldman et al., 2017). Conventional IVF-only cycles, ICSI-only cycles and IVF/ICSI mixed cycles were included and it was concluded that IVF can perfectly be applied for PGT-M with a negligible rate of sperm contamination. Similarly, a study analysing IVF or ICSI blastomeres with FISH (7 probes) was unable to detect differences in aneuploidy rate between both insemination methods (Şahin et al., 2017). However, this use of IVF/ICSI sibling oocytes has never been performed for day 5-7 PGT-A analysis.

Interestingly the mtDNA copy number is also analysed in PGT-A cycles; as trophectoderm quality and ploidy state have been described to affect the mtDNA copy number in ICSI cycles, the question remains whether this is also influenced by the insemination method.

This leaves the question if the use a of more physiological insemination method (IVF) has a beneficial impact on the ploidy state, embryo development and mtDNA content as compared to ICSI.

## Justification:

To the best of our knowledge, no study has been performed analysing the ploidy state and mtDNA content by PGT-A on IVF/ICSI sibling embryos. Also, no studies are available comparing the developmental kinetics by time lapse imaging between IVF and ICSI embryos within the same patient.

With the overuse of ICSI for non-male factor infertility, this study aims to provide evidence that conventional IVF should be the method of choice.

# Methodology

## Study Design

A pilot study will be performed on sibling oocytes comparing the effect of two insemination methods (IVF versus ICSI) on the blastocyst ploidy state.

## Study period and context

The study will start after protocol approval, and approval from the ethical committee, estimated in October 2018. Patients performing a fertility treatment at IVI Middle East Fertility Clinic, Abu Dhabi can be included if they accept to participate in the study and after signing the informed consent. It is estimated that all patients can be included in a period of three months.

Pre-implantation development, mtDNA copy number and PGT-A will be analyzed. All the aforementioned data collected for this study is obtained by usual practice.

## Reference Population

The study population is defined by all couples seeking a fertility treatment due to primary or secondary infertility in IVI RMA Abu Dhabi, for non-male factor infertility in combination with a PGT-A request.

## Subject Inclusion/exclusion criteria

*Inclusion criteria*

- Sperm parameters
  - Sperm concentration before capacitation: >15*10^6^ per ml (WHO)
    - Total motility (PR+NP,%): >40 (WHO)
    - Progressive motility (PR,%):>32 (WHO)
  - Sperm concentration after capacitation: >0.6*10^6^ per ml (not WHO defined)
    - Progressive motility (PR,%):>65 (WHO)
- ≥10 COCs after oocyte retrieval
- BMI ≤30 kg/m^2^
- Female age 18 to ≤ 40 years
- All ovarian stimulation protocols
- Fresh ejaculates
- Presence or absence of sperm morphology data: as we do not have a diagnostic sperm analysis for all patients, the presence or absence of >4% normal morphology (WHO) will not be taken into account, even with known low (<4%) normal morphology
- Couples requesting PGT-A
- Arab population

*Exclusion criteria*

- If after denudation (ICSI) only 2 mature oocytes are obtained
- If all time lapse spaces are occupied
- If the volume to be added after IVF is insufficient to perform IVF on all needed oocytes
- Presence of >1 *10^6^ per ml round cells in the ejaculate
- If a couple’s previous cycle was included in the study

## Intervention and Follow-up

Couples that are eligible for the study will be selected on the day of OPU based on all inclusion and exclusion criteria (as described under “D. Subject inclusion/exclusion criteria”) and will be asked to participate in the study by signing the IC. A randomization list will determine to which group the first half (or one extra in case an odd number is available) of the oocytes will be assigned. As to prevent selection bias, oocytes will be assigned to both groups under low magnification and different cumulus expansion stages will be evenly divided. Oocytes selected for ICSI will be denuded and injected on the day of OPU after which their pre-implantation development will be followed by time lapse imaging. IVF oocytes will be inseminated (individual or per two) overnight with 10.000 PR sperm cells in a G185 incubator (K systems) and denuded in the morning of day 1 after which they will be transferred to the time lapse imaging incubator. Blastocysts of sufficient quality (to determine this, morphokinetic parameters shall be measured) will be biopsied on day 5-7 after which the blastocysts are vitrified individually. PGT-A (NGS platform) is performed on all TE biopsy samples and the ploidy state and mtDNA score will be provided. All annotated time lapse imaging data will be gathered and combined with the pre-implantation data, PGT-A data (ploidy state and mtDNA copy number) and all general patient and cycle characteristics. Patients with euploid embryos available will receive a FET and implantation rates and (clinical) pregnancy outcomes will be recorded.

The follow-up of the patients does not deviate from a normal fertility trajectory.

Quantification of the determining variables:

The primary outcome variable is the ploidy state

Secondary outcome variables are the (i) embryo development defined by the number of COCs retrieved, maturation and fertilization rate, embryo development analysed on day 3 and day 5, total number of embryos used for the patient, day of biopsy and the morphokinetic parameters; (ii) mtDNA copy number and (iii) the clinical outcome.

# Statistical methodology

## Data base

The database will be a description of:

- data acquisition platform
- registration and use platform, for analysis
- definition of the structure of the database, if applicable

The database will be rigorously defined with the variables destined to be analyzed according to the objectives set. The necessary information will be exported from the clinical information manager, SIVIS, to a table in Excel format through a database-based query system.

The exported data will be duly codified in order to protect the clinical and personal information of the patients according to the applicable law in the place where the research project is carried out.

Finally, and prior to the statistical study, an exploratory data analysis will be carried out to review the quality of the information extracted.

## Study Variables

### Main-outcome measures (Dependent)

PGT-A:

- Ploidy outcome: euploid or aneuploid

### SECONDARY outcome measures (Dependent)

PGT-A:

- mtDNA copy number (de los Santos et al., 2018):
  - Mitoscore value: represents the normalized mtDNA content in (an)euploid embryos and indicates the total mtDNA content in the sample

Pre-implantation development:

- Number of COCs retrieved
- Maturation rate: number of mature oocytes obtained in each arm
  - ICSI: defined on day 0 as the number of injected oocytes/number of COCs assigned to the ICSI group
  - IVF: defined on day 1 as the number of oocytes with an extruded PB/number of inseminated oocytes
- Fertilization rate: number of zygotes with 2PN/number of oocytes assigned to the group
- Embryo quality on day 3 defined by the Istanbul consensus (Alpha Scientists, 2011)
  - The number of blastomeres and their division pattern, fragmentation, presence of compaction, vacuoles, granulation and nuclei will divide the embryos into 3 categories: good, fair or poor
  - Cell number on day 3 for all fertilized oocytes
- Embryo quality on day 5 (Gardner and Schoolcraft,1999) defined by the Istanbul consensus (Alpha Scientists, 2011)
  - The blastulation rate: number of embryos reaching at least the BL1 stage/ number of oocytes assigned to that group: defined by a 1 (yes, the embryo is blastulating) or 0 (no, the embryo is not blastulating)
  - The expansion stage of the blastocyst and the quality of the ICM and TE will divide the embryos into 3 categories according to Istanbul consensus
- Utilization rate: number of embryos that can be used for the patient (=number of embryos that can be biopsied)/number of oocytes assigned to the group
- Day on which the biopsy is performed (day 5,6 or 7)
- Morphokinetic parameters defined by time lapse imaging starting from the time of syngamy/PN fading (Basile et al., 2014)
  - t2: the timing to two cells
  - t3: the timing to three cells
  - t4: the timing to four cells
  - t5: the timing to five cells
  - t8: the timing to eight cells
  - SC: starting to compact
  - M: the timing to morula
  - SB: starting to blastulate
  - B: reaching a BL3 according to Gardner and Schoolcraft, 1999
  - F: reaching a BL4 according to Gardner and Schoolcraft, 1999
  - cc2: t3-t2
  - s2: t4-t3
- Biopsy yes or no: indicating developmental competence or developmental arrest
- Blastocyst gender: male or female

Clinical cycle outcome:

- Pregnancy (yes or no) defined by a βhCG test of > 15IU
- Biochemical pregnancy (yes or no) defined as a pregnancy in which the hCG levels start do decrease after 1 week
- Implantation rate (%) defined by the number of gestational sacs/number of embryos transferred
- Clinical pregnancy (yes or no) defined by the ultrasonographic visualization of one or more gestational sacs, including ectopic pregnancies

### explanatory variables (INDEPENDENT)

Principal exposure factor

- Intervention: IVF or ICSI

Patient and cycle characteristics

- Sperm parameters before and after capacitation:
  - Concentration
  - PR
- BMI (kg/m^2^)
- Female age at the moment of OPU (years)
- AMH
- Smoking habit
  - Not smoking: 0
  - Smoking: 1
- Race
- Hormones on the day of trigger
  - E_2_ (pg/ml)
  - P (ng/ml)
- Ovarian stimulation
  - Ovarian stimulation protocol
  - Total dose of gonadotrophins used (IU)
  - Duration of stimulation (days)
- Trigger
  - Type
  - Units (IU)
- Abstinence duration
- Indication for PGT-A testing
- Endometrial preparation for FET
- Primary or secondary infertility

## Sample Size

Considering no study has been performed analyzing the ploidy state, mtDNA content and embryo morphokinetics by PGT-A on IVF/ICSI sibling embryos, we propose a pilot study including 30 couples to assess the feasibility of the study. Moreover, the pilot study will allow us to estimate statistical parameters which enables us to calculate a sample size in a future study in which an acceptable level of power can be established.

## Statistical data analysis

### Exploratory data analysis

Summary about data collected. Categorical data will be presented in frequency tables and histograms. Continuous values will be summarized according the mean, standard deviation and confidence interval.

Representation of categorical data through bar plots and continuous data through boxplots or density plots.

In other way, it will allow evaluating data quality and detecting anomalies (blank data, outliers, etc).

### Homogeneity analysis

Although the selection criteria and study design tend to preserve the homogeneity of the study groups, it is important to ensure that there are no external population effects. Therefore, the control variables mentioned above will be compared in order to assess the comparability of the groups.

Categorical variables will be compared using a Chi-square test, and for quantitative variables, a t-Student test (assuming normality, if it is not possible to assume it, a Mann-Whitney test will be applied).

### Objectives assessment

Main objective

In order to compare the effect of IVF versus ICSI on the blastocyst ploidy state a Poisson regression will be performed, which is the most appropriate statistical modelling method to analyse count data.

Secondary objectives

To compare IVF versus ICSI outcomes in preimplantation development outcomes:

- To compare the maturation rate, a t-test will be used to detect differences in the average number of mature oocytes (a Wilcoxon test will be used if we cannot assume normal distribution of data). If we found differences, linear or Poisson regressions will be performed to evaluate them in a model taking into account rest of explanatory variables.
- To compare the fertilization rate, a t-test will be used to detect differences in the average number of zygotes with 2PN oocytes (a Wilcoxon test will be used if we cannot assume normal distribution of data). If we found differences, linear or Poisson regression will be performed to evaluate them in a model taking into account rest of explanatory variables.
- In order to compare embryo quality on D3 (measured using the Istanbul consensus), a multinomial logistic regression will be performed.
- Embryo quality D5
  - To compare the blastulation rate, a t-test will be used to detect differences in the average number of embryos reaching at least the BL1 stage (a Wilcoxon test will be used if we cannot assume normal distribution of data). If we found differences, linear or Poisson regressions will be performed to evaluate them in a model taking into account rest of explanatory variables.
  - In order to compare embryo quality on D5 (The expansion stage of the blastocyst and the quality of the ICM and TE will divide the embryos into 3 categories), a multinomial logistic regression will be performed.
- To compare the utilization rate, a t-test will be used to detect differences in the number of embryos that can be used for the patient (a Wilcoxon test will be used if we cannot assume normal distribution of data). If we found differences, linear or Poisson regressions will be performed to evaluate them in a model taking into account rest of explanatory variables.
- In order to evaluate morphokinetic parameters defined by time-lapse imaging starting from the time of syngamy and their effect on ploidy, biopsy day, embryo arrest and gender, first we will present a trend chart to describe the average development times of the embryos according to the treatment. Then, growth models (linear or exponential) will be performed for each embryo, to estimate a growth parameter that can be compared between IVF and ICSI treatments.

To compare IVF versus ICSI outcomes in mtDNA:

- A t-test will be used to detect differences in the Mitoscore value depending on the treatment (IVF or ICSI) if we can assume normality distribution for Mitoscore value (in other case, a Wilcoxon test will be performed). Moreover, linear regression models will be performed to analyse the treatment effect in the presence of the remaining covariates.

To compare IVF versus ICSI outcomes in cycle outcomes:

- We will build a contingency table, and then, a chi-squared test will be used in order to compare the pregnancy, biochemical pregnancy, and clinical pregnancy results between IVF versus ICSI treatments. If we found statistical differences, a logistic regression will be performed to evaluate this differences in a model taking into account rest of explanatory variables.
- To compare the implantation rate, a t-test will be used to detect differences in this rate (a Wilcoxon test will be used if we cannot assume normal distribution of data). If we found differences, linear or Poisson regressions will be performed to evaluate them in a model taking into account rest of explanatory variables.

# Work plan

Phase I: submission of the Biomedical Research protocol and obtaining approval and, submission (and approval) by the local ethical committee

Phase II: start of the study: including the patients

- Embryologists:
  - selecting patients that are eligible for the study in consultation with the responsible physician
  - annotating in the time lapse system
  - performing biopsy and sending the samples
- Study nurse: explaining patients about the study and obtain the IC
- Responsible physician: follow-up of the patients included
- PGT-A and mtDNA copy numberanalysis

Phase III: preparing the database

- Embryologist:
  - Make the database with extraction from SIVIS
  - Double check (with 2 embryologists) all annotations
  - Check if all the data is present in the database
  - Clean the database

Phase IV: statistics

- Statistician:
  - Perform necessary tests
  - Discuss data with embryologists and responsible physician where needed

Phase V: write paper

- Embryologists and responsible physician:
  - Write manuscript
- Statistician:
  - Write part about statistics
  - Check interpretations (results and statistical outcome)
- All other parties involved:
  - Review the manuscript

|  | 2018 | | | | | | | | | | | |  |
| --- | --- | --- | --- | --- | --- | --- | --- | --- | --- | --- | --- | --- | --- |
|  | Jan | Feb | Mar | Apr | May | Jun | Jul | Aug | Sep | Oct | Nov | Dec | |
| Phase I |  |  |  |  |  |  |  |  |  |  |  |  | |
| Phase II |  |  |  |  |  |  |  |  |  |  |  |  | |
| Phase III |  |  |  |  |  |  |  |  |  |  |  |  | |
| Phase IV |  |  |  |  |  |  |  |  |  |  |  |  | |
| Phase V |  |  |  |  |  |  |  |  |  |  |  |  | |
|  |  |  |  |  |  |  |  |  |  |  |  |  | |
|  | 2019 | | | | | | | | | | | |  |
|  | Jan | Feb | Mar | Apr | May | Jun | Jul | Aug | Sep | Oct | Nov | Dec | |
| Phase I |  |  |  |  |  |  |  |  |  |  |  |  | |
| Phase II |  |  |  |  |  |  |  |  |  |  |  |  | |
| Phase III |  |  |  |  |  |  |  |  |  |  |  |  | |
| Phase IV |  |  |  |  |  |  |  |  |  |  |  |  | |
| Phase V |  |  |  |  |  |  |  |  |  |  |  |  | |

# Ethical issues

This Research Project respects the fundamental principles of the Declaration of Helsinki, the Council of Europe Convention on Human Rights and Biomedicine, the UNESCO Universal Declaration on the Human Genome and Human Rights, as well as the requirements of Spanish law in the field of biomedical research, the protection of personal data and bioethics.

For the inclusion of all patients in the study, it is needed signed informed consent, which is approved by the Ethics Committee from the IVI RMA Fertility Clinic, Abu Dhabi, research centre.

# Funding

No funding should be obtained for this study as no extra costs are needed for the IVF lab to perform this study.

| Needs funding | No | | | |
| --- | --- | --- | --- | --- |
| Externally Funded project | No | Funding Institution | | Not Applicable |
| External Funding plan (if no funding is available yet) | NA | | | |
| IVIRMA Funding requested | NA | Internally Funded project | | No [To be filled after the SAB decision has been made available] |
| Funding time range | Start: {date} | | Finish: {date} | |
| Budget | {Budget} | | | |

# ANNEX 1. Estimation of the research Project marginal costs

Participation to this study does not generate extra activities on top of the usual practice, nor does it generate an extra cost for the center. This means that no extraordinary activities are performed and that the patient will be treated without any deviation from her normal treatment.

# Insurance

IVI RME Fertility, Abu Dhabi, UAE has an Insurance Policy in force that conforms to current legislation and with coverage to compensate and indemnify cases of ill health or injury, which may arise in connection with their participation in the study, within routine clinical practice.

# Publication and diffusion

Human Reproduction: IF 4.990

Fertility and Sterility: IF 4.803

ESHRE 2019 if feasible, otherwise ASRM 2019

- Oral: PGT-A, mtDNA and morphokinetics in IVF versus ICSI

# References

Alpha Scientists in Reproductive Medicine and ESHRE Special Interest Group of Embryology (2011) The Istanbul consensus workshop on embryo assessment: proceedings of an expert meeting. Hum Reprod 26:1270-1283.

Basile N, Vime P, Florensa M, Aparicio Ruiz B, García Velasco JA, Remohí J, Meseguer M (2014) The use of morphokinetics as a predictor of implantation: a multicentric study to define and validate an algorithm for embryo selection. Hum Reprod 30(2):276-83.

Boulet SL, Mehta A, Kissin DM, Warner L, Kawwass JF, Jamieson DJ (2015) Trends in use of and reproductive outcomes associated with intracytoplasmic sperm injection. JAMA 313:255-263.

de Los Santos MJ, Diez Juan A, Mifsud A, Mercader A, Meseguer M, Rubio C, Pellicer A (2018) Variables associated with mitochondrial copy number in human blastocysts: what can we learn from trophectoderm biopsies? Fertil Steril 190(1);110-117.

Dyer S, Chambers GM, de Mouzon J, Nygren KG, Zegers-Hochschild F, Mansour R, Ishihara O, Banker M, Adamson GD (201) International Committee for monitoring assisted reproductive technologies world report: assisted reproductive technology 2008, 2009and 2010. Hum Reprod 31:1588-1609.

Feldman B, Aizer A, Brengauz M, Dotan K, Levron J, Schiff E, Orvieto R (2017) Pre-implantation genetic diagnosis-should we use ICSI for all? J Assist Reprod Genet 34(9):1179-1183.

GardnerDK and Schoolcraft WB (1999) In vitro culture of human blastocysts. In: Jansen R, Mortimer D (eds). Toward Reproductive Certainty: Fertility and Genetics Beyond. UK: Parthenon Publishing London,378-388.

Li Z, Wang AY, Bowman M, Hammarberg K, Farquhqr C, Johnson L, Safi N, Sullivan EA (2018) ICSI does not increase the cumulative live birth rate in non-male factor infertility. Hum Reprod 33(7):1322-1330.

Şahin L, Bozkurt M, Şahin H, Gürel A, Cahskan E (2017) To compare aneuploidy rates between ICSI and IVF cases. Niger J Clin Pract 20(6):652-658.

Van Landuyt L, De Vos A, Joris H, Verheyen G, Devroey P, Van Steirteghem A (2005) Blastocyst formation in in vitro fertilization versus intracytoplasmic sperm injection cycles: influence of the fertilization procedure. Fertil Steril 83(5):1397-403.

Zegers-Hochschild F, Adamson GD, Dyer S, Racowsky C, de Mouzon J, Sokol R, Rienzi L, Sunde A, Schmidt Lm Cooke ID, Simpson JL, van der Poel S (2017) The international glossary on infertility and fertility care, 2017. Hum Reprod 32(9):1786-1801.
